# Understanding crisis dynamics during public health emergencies: an integrated framework of social consequences, trust, and behavioral responses

**DOI:** 10.3389/fpubh.2026.1875500

**Published:** 2026-07-15

**Authors:** Wenyue Xu, Naizhe Li

**Affiliations:** 1 Joint International Research Laboratory of Catastrophe Simulation and Systemic Risk Governance, Beijing Normal University, Zhuhai, China; 2 Beijing Research Center for Respiratory Infectious Diseases, Beijing Key Laboratory of Surveillance, Early Warning and Pathogen Research on Emerging Infectious Diseases, School of National Safety and Emergency Management, Beijing Normal University, Beijing, China

**Keywords:** infodemic, public health emergencies, social consequences, social resilience, trust erosion

## Abstract

Public health emergencies are inherently social crises that disrupt institutions, livelihoods, and everyday social functioning. However, the factors that determine whether their crisis dimensions are amplified, sustained, or attenuated remain insufficiently understood from an integrated, macro-systemic perspective. This review synthesizes evidence across epidemiology, sociology, psychology, and public health to examine the interacting mechanisms that shape the social dynamics of epidemics. We develop a framework in which social consequences, trust dynamics, behavioral and psychological responses, and information environments jointly influence crisis trajectories through reciprocal feedback processes. Trust is conceptualized as a critical mediating factor linking governance to public behavior, while inequalities and information environments condition whether disruptions are contained or amplified. We conclude that epidemic preparedness should move beyond a pathogen-centered model to integrate social protection, trust-sensitive communication, mental health support, and infodemic management.

## Introduction

Public health emergencies are increasingly recognized as systemic shocks that affect governance, economic activities, social interactions, and collective psychological well-being (1–3). Existing scholarship has long argued that epidemics are not merely biomedical phenomena but are fundamentally social events (4, 5). The COVID-19 pandemic reshaped work, education, access to care, food security, social relations, and daily mobility, causing people to feel greater distress and worsening social inequality (6). The pandemic also revealed how preexisting weaknesses in preparedness, governance, and public trust could worsen these broader consequences, as countries with stronger institutional legitimacy and social cohesion usually performed better than preparedness metrics alone would predict (7, 8).

The COVID-19 pandemic also showed important variation in how the crisis dimensions of epidemics unfold across societies. Epidemic control highly depends on compliance, cooperation, leadership, and communication (9–11). Such control measures are especially important in prolonged crises, during which the sustainability of restrictive measures depends not only on risk perception but also on whether policies are experienced as fair, intelligible, and collectively meaningful (10, 12). Risk communication, trust, and negative emotions shape both protective and excessive behavioral responses during crises (13). Moreover, digital and social media environments are crucial in this process: they enable early warning, public feedback, and policy adaptation but also contribute to panic, stigma, misinformation, and social division (14–16). Information overload and conflicting narratives could become part of the crisis environment by undermining trust in institutions and weakening the public's ability to act on verified health guidance (17, 18). These observations suggest that the social trajectories of epidemics are shaped by interacting institutional, behavioral, informational processes rather and epidemiological dynamics.

Several factors have been extensively studied, yet this evidence remains dispersed across disciplinary boundaries (13, 19, 20). The way in which these components combine to produce broader social instability remains poorly understood. Visible unrest might be reduced in the short term because of mobility restrictions and emergency controls, but social strain can accumulate and reemerge as distrust, grievance, and instability once the immediate health threat is (21, 22). Evidence from the United States further suggested that pandemic-related unemployment and mortality intensified negative emotional stress and deteriorated economic perceptions, which were associated with greater levels of social unrest (23). Declining wellbeing, loneliness, and unequal social burden are concentrated in already vulnerable groups, indicating that epidemics produce crises by interacting with and deepening existing inequities (6, 24, 25). This gap is important because it hides the shared patterns behind various outbreaks and restricts the development of integrated preparedness strategies.

While previous reviews have documented the social and behavioral consequences of epidemics, less attention has been paid to how social consequences, trust dynamics, behavioral responses, and information environments interact through feedback processes to shape crisis trajectories. This review aims to explore an integrated mechanism framework for understanding the crisis dynamics of public health emergencies. We define the crisis dimensions of epidemics as disruptions to the normal functioning of social institutions and collective life, including livelihoods, education, healthcare access, social relations, political legitimacy, and social cohesion. These dimensions may manifest as inequality, distrust, stigma, polarization, collective unrest, or other forms of social instability. We conceptualize epidemics as social crises whose trajectories are shaped by feedback interactions among social consequences, trust dynamics, behavioral and psychological responses, and information environments. These mechanisms interact recursively, with social consequences influencing trust, trust shaping behavioral responses, and information environments amplifying or attenuating these effects over time. We argue that trust serves as a critical mediating mechanism linking governance to public behavior. In doing so, we hope to provide a public health account of why the social crisis dimensions of epidemics are amplified, sustained, or attenuated across different social and institutional contexts.

## Literature search and study selection

This review was conducted as a structured narrative review rather than a formal systematic review. Literature was identified through searches of PubMed, Web of Science, and Google Scholar covering the period from January 1, 2000, to January 1, 2026. Search terms combined concepts related to epidemics and public health emergencies (“pandemic,” “epidemic,” “infectious disease outbreak,” “COVID-19,” “Ebola,” “SARS,” and “Zika”) with terms related to social consequences (“inequality,” “social disruption,” “stigma,” “polarization,” “social unrest”), trust processes (“trust,” “institutional trust,” “risk perception,” “risk communication”), and information dynamics (“misinformation,” “infodemic,” “social media,” “information environment”).

Studies were selected based on their relevance to one or more components of the proposed framework, including social consequences, trust dynamics, behavioral and psychological responses, information environments, and collective instability. Priority was given to peer-reviewed empirical studies, systematic reviews, influential theoretical contributions, and major international reports that provided evidence relevant to epidemic crisis dynamics. Additional studies were identified through backward and forward citation tracking of key articles.

## Conceptual framework

**Figure 1:**
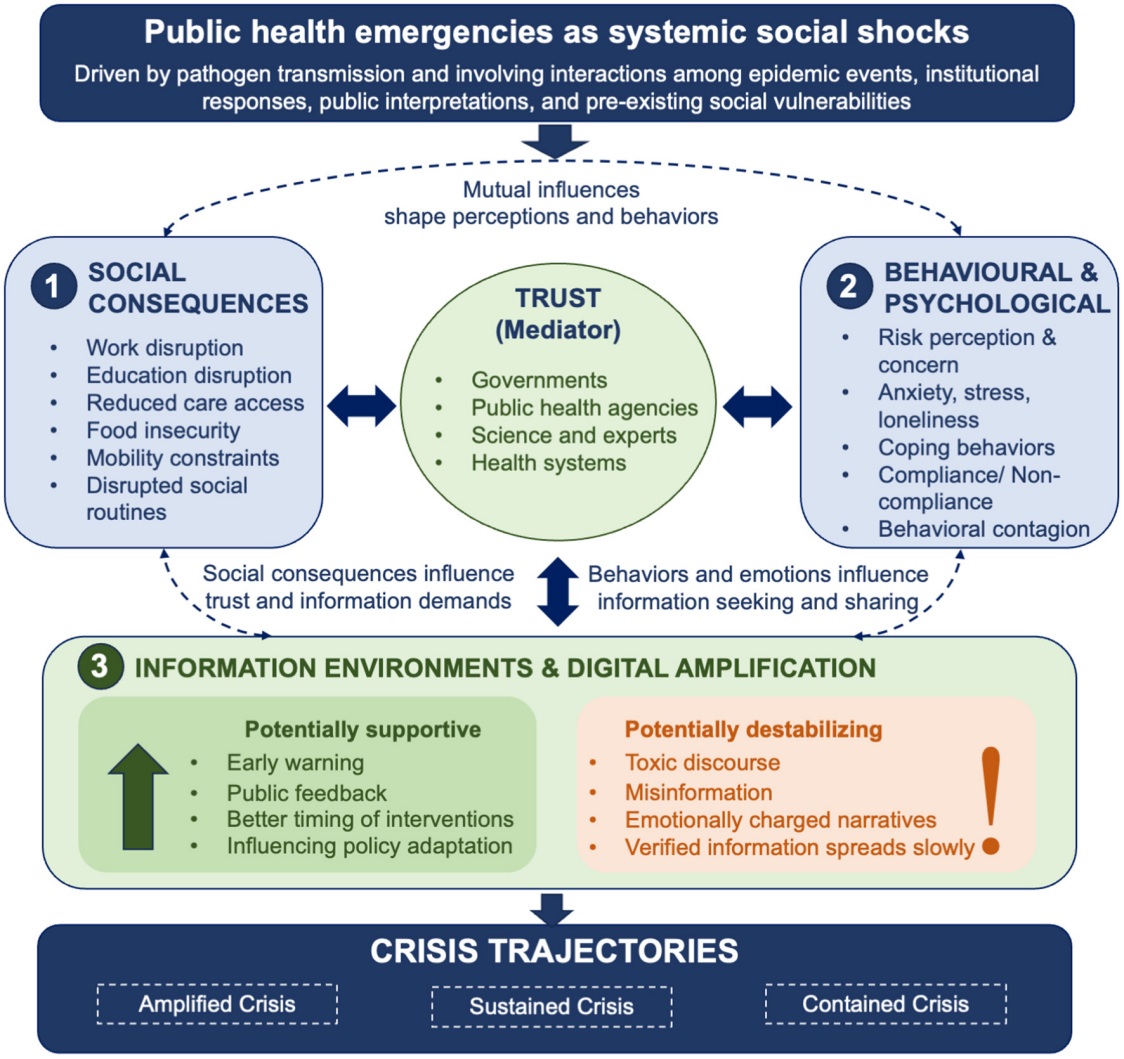
Integrated mechanism framework of crisis dynamics during public health emergencies.

We conceptualize public health emergencies as systemic social shocks, which are caused by pathogen transmission and involve interactions among epidemic events, institutional responses, public interpretations, and preexisting social vulnerabilities (26). In this framework (Figure 1), we propose that social consequences, trust dynamics, behavioral and psychological responses, and information environments interact through reciprocal feedback processes that shape how epidemics unfold within society. This framework is consistent with the pandemic risk management scholarship, which argues that effective response requires balancing expert assessments of actual risk with public perceptions of risk, while integrating social, economic, and environmental determinants into crisis governance (27, 28).

In this framework, social consequences constitute one of the core dimensions through which epidemic crises are experienced. Disruptions during public health emergencies such as in work, education, care access, food security, mobility, and social routines are filtered through existing inequalities. During the COVID-19 pandemic, social and economic burdens were concentrated in already disadvantaged groups, including children, low-income households, migrants, and racially marginalized communities (24, 25). Therefore, epidemics are better understood as events that expose and deepen structural inequities, which creates the conditions under which crises move beyond the health sector and begins to affect wider social stability.

Trust occupies a central position within the framework because it mediates the relationship between governance and public behavior. The effectiveness of epidemic control hinges on populations' perceptions of institutions as credible, legitimate, and responsive. Compliance with interventions was shaped by trust in the government, perceptions of uncertainty, and a willingness to cooperate under conditions of disruption (9, 29). In our framework, trust is treated as a critical mediating factor that connects governance to public behavior. It is simultaneously shaped by social consequences and capable of reshaping them. Whereas trust might be strengthened by clear and consistent communication, it might be undermined by delayed messaging, visible inconsistency, or perceived unfairness in the distribution of burdens (11).

Behavioral and psychological responses represent another interacting component of crisis dynamics. Public health emergencies cause psychological distress, which affects people's interpretation of information and their response to policy (30). Compared with objective social isolation alone, cross-cultural evidence from the USA, South Korea, France, and Hong Kong indicates that during the early phase of the pandemic, psychosocial distress was more consistently linked to loneliness, concern about the pandemic, and the surrounding social context (31). These responses are shaped by levels of trust and by the unequal distribution of social consequences, yet they also feed back into the crisis process (24). Psychological distress may increase susceptibility to misinformation, reduce willingness to cooperate, and intensify social tensions. Conversely, social solidarity, resilience, and collective efficacy may help buffer crisis pressures and support adaptive responses.

Information environments constitute a further mechanism through which crisis dynamics are amplified or moderated. Digital platforms can support early warning and enhance public health intervention timing, but research has shown that during the COVID-19 pandemic, online sentiment might influence policy adaptation (14, 18, 32). Similarly, in such environments, toxic discourse, misinformation, or emotionally charged narratives spread more rapidly than verified public health communication does (16, 33).

A key contribution of this framework is its emphasis on feedback processes. Crisis escalation occurs when a series of strains accumulate and strengthen each other over time. Unequal social burdens may erode trust; declining trust may reduce compliance; reduced compliance may prolong disruption; and prolonged disruption may further deepen social inequalities. Similar feedback processes may occur between information environments and trust, or between psychological strain and collective behavior. These interactions help explain why epidemic crises follow different trajectories across societies and why similar epidemiological threats can produce markedly different social outcomes.

## Social consequences as a core dimension of epidemic crises

Social consequences represent one of the most visible and widely experienced dimensions of epidemic crises. During the COVID-19 pandemic, restrictions on movement, school closures, workplace shutdowns, disrupted care pathways, and reduced access to routine services reshaped everyday life at the population level (25, 34). These disruptions altered how households generated income, how children learned, how people accessed food and health care, and how social support was organized (24). These social consequences were part of the crisis.

The consequences are cumulative and unequal. Public health emergencies tend to magnify preexisting social gradients in terms of exposure, protection, and recovery (35). Low-income households, precarious workers, migrants, racially marginalized communities, and families with limited access to social protection are less able to absorb sudden disruptions in work, schooling, and care (21, 24). Therefore, the social burden of epidemics is patterned by existing inequality rather than being randomly distributed across the population. This pattern was particularly visible during multiple outbreaks, including COVID-19, Ebola, and Zika, especially in communities that had already experienced structural disadvantage (24, 36–38).

Children and women clearly illustrate this pattern. The indirect effects of the pandemic on children extended beyond missed schooling to worsening educational inequality, family conflict, social isolation, and heightened long-term risk associated with household economic deterioration (39). Evidence from low-income settings suggested that pandemic conditions were associated with high levels of intimate partner violence, indicating that social crisis may be translated into violence within households as well as in public spaces (40).

Social consequences occurred with a time lag, which helps explain why the social effects of epidemics may outlast their acute biomedical phase. Historical work on the 1889–1890 influenza pandemic suggests that epidemic disruption can become entangled with wider political, economic, and cultural instability in ways that persist beyond the period of peak transmission (3). Comparable dynamics have been observed following Ebola outbreaks, where disruptions to livelihoods, education, and trust in institutions persisted even after epidemiological control had been achieved (37). Social consequences should be understood as a process of social sedimentation, in which repeated disruptions accumulate gradually into wider instability. These consequences become politically important not only because they cause hardship but also because they change how populations see the fairness, competence, and legitimacy of institutions managing crises.

## Trust as the key mediating factor of crisis dynamics

Trust is the key mediating mechanism through which a public health emergency moves from social disruption to broader societal instability. Here, trust refers primarily to institutional trust, including trust in governments, public health authorities, scientific expertise, and crisis management institutions. Disruptions in work, mobility, schooling, and care may remain governable in one setting but generate resistance, anxiety, and grievances in another. A central difference is whether populations continue to see institutions as credible, fair, and capable under conditions of uncertainty. Trust was closely connected to the implementation of government measures, public compliance, and perceptions of legitimacy, making it more than a background condition for crisis response. Trust functions as the social infrastructure that links governance to behavior (11, 41, 42). Evidence during the 2018–2020 Ebola outbreak in the Democratic Republic of the Congo suggests that trust varied substantially across institutions and strongly influenced willingness to participate in surveillance, vaccination, and treatment programs, highlighting the central role of institutional legitimacy in epidemic governance (43).

Trust in epidemics is not a single construct. In China, greater exposure to the risks associated with COVID-19 reduced general and political trust significantly overall, whereas the effects on the trust of physicians and local officials were more conditional (44). In Canada, socioeconomic position influenced significantly the decrease in trust of individuals who experienced risks associated with COVID-19 (45). Confidence and trust during the Australian COVID-19 response varied across institutions and were patterned by demographic and social factors rather than moving uniformly across the population (46). These findings suggest that epidemics influence trust relations across society, creating uneven capacities for cooperation, resilience, and collective action.

Trust could shape how risk is interpreted. Public trust in science during the COVID-19 pandemic depended on both the content of expert advice and how scientific uncertainty was communicated and politically contested (47). In China, trust in local government and media was associated with lower COVID-19 infection rates, and these relationships were mediated wholly or partly by risk perception (48). In South Korea, lower levels of trust in the government were associated with higher levels of affective and cognitive risk perception across a year-long national study (49). In Switzerland, declining social trust and lower perceived risk levels were linked to reduced acceptance of public health measures over time (12). These findings reveal that when trust weakens, uncertainty can be processed as suspicion, inconsistency, or threat, which can undermine adherence even when formal measures remain in place.

Trust erosion is affectively consequential. Trust, risk perception, worry, and adherence can shift rapidly and alter the trajectory of crisis response (50). In China, satisfaction is correlated negatively with government management and public anxiety, and this relationship is partially influenced by risk perception (51). Other studies have shown that risk communication, trust, risk perception, and negative emotions are interlinked in shaping both protective and excessive behavioral responses (13). This relationship helps explain why the erosion of trust can intensify crises even before overt unrest emerges: when institutions lose persuasive authority, the public is more likely to interpret uncertainty through anxiety, anger, and reactive behavior rather than cooperative restraint.

The consequences of trust can persist during the post-outbreak period. Research in Africa has shown that individuals who have been exposed to infectious disease outbreaks have shown a measurable decrease in their trust in political institutions (52). Similarly, qualitative work from post-Ebola Liberia revealed that distrust of government and denial of the epidemic persisted years after the emergency, despite some positive behavioral changes remaining (53). These studies suggest that even when transmission declines, trust deficits can remain embedded in political memory and community interpretation, weakening later preparedness and making future crises more difficult to govern.

Taken together, the evidence suggests that trust is best understood as a dynamic mediator of crisis trajectories. By connecting governance, risk perception, behavioral responses, and institutional legitimacy, trust helps explain why similar epidemiological threats may generate markedly different social outcomes across populations and over time.

## Behavioral and psychological processes through which tensions intensify

Behavioral and psychological processes are the mechanisms through which instability is expressed. Public health emergencies heighten concern, uncertainty, loneliness, and emotional strain (30). Anxiety, depression, trauma-related symptoms, and loneliness then likely to become major secondary burdens of the crisis, especially under conditions of quarantine and prolonged uncertainty, such as those that occurred during the COVID-19 pandemic (19, 54). Compared with objective social isolation alone, psychological distress during the initial phase of the COVID-19 pandemic was associated more consistently with loneliness, concern about the pandemic, and the surrounding social context (31). Expressed sentiment declined globally and significantly during the COVID-19 outbreak, followed by a slower and more uneven recovery, indicating that psychological strain also unfolded as a population-level emotional process (55). This process shapes how people interpret risk, how they evaluate official guidance, and whether they respond with cooperative restraint, withdrawal, or reactive behavior.

Not all of these responses lead to destabilization. Greater concern could be associated with greater uptake of self-protective behaviors, while this relationship is strongly conditioned by emotion, trust, and the social meaning of risk (49). When a concern is tied to psychological stress, it might produce behaviors that are individually rational in the short term but socially destabilizing in the aggregate. Research has shown that compared with epidemiological indicators alone, anxiety and depressive symptoms are more strongly associated with concerns and quarantine behaviors related to COVID-19, suggesting that subjective psychological responses may shape everyday action more directly than objective disease metrics do (56).

Material insecurity sharpens this process. Financial strain, employment loss, and disrupted routines could add to the psychological burden and help convert distress into socially consequential behavior. Employment loss was associated with higher levels of depressive symptoms and COVID-19-related concern, whereas university students reported that financial strain and psychological distress were linked to unhealthy coping behaviors and deterioration in daily self-regulation (56, 57). These findings reveal how economic insecurity can transform mental distress into household and community-level instability.

Panic purchasing and other types of behavioral contagion are clear manifestations of this phenomenon. During the COVID-19 pandemic, panic buying was repeatedly linked to uncertainty, perceived scarcity, anxiety, and social media exposure (58, 59). Risk perception and state anxiety influenced each other, while trust in social media also contributed to the tendency to follow others in terms of queueing and over-purchasing behavior (59). Platforms can also create panic by rapidly circulating emotionally charged images, rumors, and narratives of shortage, thereby converting private fear into public behavioral cascades (60). These behaviors illustrate that once emotions are mirrored and amplified in real time, they can strain supply systems, heighten perceptions of insecurity, and weaken confidence in institutional control.

Digital information environments intensify these mechanisms further by amplifying emotionally salient misinformation. Misinformation on social media can spread effectively when it is framed around emotionally charged themes such as threat, corruption, treatment controversy, or social grievances (61, 62). Misinformation was amplified through specific themes and engagement patterns, whereas reviews of COVID-19 misinformation noted that the volume and circulation of false claims undermined public health communication and intensified confusion (61, 62). This finding indicates that behavioral escalation can influence how digital environments reward emotionally provocative content, especially under conditions of low trust and high uncertainty.

These findings suggest that behavioral and psychological processes intensify societal tension through three interlocking pathways. First, loneliness, anxiety, and chronic concern reduce emotional resilience and increase the salience of threats. Second, financial strain and disrupted routines make these emotional responses more difficult to absorb and more likely to shape daily behavior. Third, digital and social media environments amplify both emotional cues and behavioral contagion, allowing private distress to become a visible collective reaction. Under conditions of weak trust and unequal burden, psychological strain becomes more likely to spill into forms of behavior that cause crisis escalation.

## Collective instability, polarization, and crisis dynamics

Collective instability represents one possible trajectory through which epidemic crises may evolve. Pandemic restrictions may intensify grievances by disrupting work, mobility, and daily life while altering opportunities for mobilization by concentrating discontent around visible state actions such as lockdowns, mandates, and closures (63, 64). On a global scale, pandemic-related disorder events were temporally clustered and self-exciting, suggesting that they could diffuse and reinforce one another over time (63). These findings show that collective instability is a dynamic process through which policy burdens, emotional responses, and event contagion interact (64, 65). However, whether crisis pressures are translated into protest, polarization, stigma, or institutional conflict depends on how social consequences, trust dynamics, behavioral responses, and information environments interact over time. Collective instability therefore emerges not from any single mechanism but from the accumulation and mutual reinforcement of multiple social processes.

One route through which this transition occurs is the politicization of hardship. Measures designed to control transmission can be experienced as unequal, coercive, or selectively enforced, especially when trust is already weak. Cross-national evidence shows that restrictions affecting workplaces, schools, movement, and gatherings were associated positively with higher levels of COVID-19-related unrest, whereas economic support policies attenuated some of these effects (64). In the United States, pandemic-related unemployment and mortality intensified negative emotional stress, and this stress was more strongly associated with unrest in politically polarized and economically strained settings (23). More broadly, pandemics can aggravate preexisting inequalities and bring them into sharper political focus, especially in contexts in which health, income, and social protection are already unevenly distributed (8). Social tensions become collectively unstable when those losses are interpreted through conflictual political frames and experienced as evidence of institutional unfairness or neglect.

The second route is the social production of blame, stigma, and moral boundary-making. Epidemics could generate stigma, attaching not only to racialized or nationalized groups but also to travelers, health care workers, dissenters, and those judged to be insufficiently compliant (66). Vaccinated individuals in multiple countries expressed discriminatory attitudes toward the unvaccinated at levels comparable to attitudes commonly directed toward immigrant or minority groups (67). Research on COVID-19-related stigma in Canada has shown that blame and moralization divided the population into “virtuous” and “immoral” groups, thereby reinforcing social control but also deepening polarization (66). Earlier work on the U.S. Ebola communication crisis similarly revealed that compared with accurate messages, misinformation was more likely to be political, discord-inducing, and risk-elevating, illustrating how fear-inducing outbreaks can quickly become communication crises as well as health crises (68). Similar patterns emerged during the Zika epidemic, where women reported that misinformation, rumors, and longstanding distrust of governmental institutions weakened confidence in official health guidance and contributed to perceptions of vulnerability and social division, highlighting how epidemic crises are shaped not only by biological risk but also by contested information environments (69). In this sense, collective instability is fueled not only by deprivation but also by narratives that convert uncertainty into accusation and behavioral difference into social threat.

A third route is information-driven amplification. Information disorder can alter the emotional climate, intensify group segmentation, and make collective reactions more volatile even when corrective communication is present. False narratives could be amplified preferentially by specific themes and engagement dynamics, while modeling work from Japan demonstrated that corrective information, if excessively diffused, could itself worsen disruption by stimulating overreaction (61, 70). Information overload, misinformation, and information voids can distort risk perception, undermine trust, and worsen compliance (17, 71). As the pandemic progressed, information overload, conflicting interpretations, and fatigue contributed to a growing perception of societal polarization (72).

The final mechanism is the reorganization of social ties. Remote communication during the COVID-19 pandemic increased interactions with politically similar others, amplifying homophily among nonkin ties during the early phase of the pandemic (73). Behavioral strain becomes more likely to crystallize into collective instability when four conditions align: burdens are widely felt, trust is eroded, information environments amplify conflict, and social networks become more segmented. When these processes coincide, public health emergencies cease to be governed primarily as health crises and begin to operate as wider crises of legitimacy, solidarity, and social order (74).

## Implications for public health governance and social resilience

Preparedness for public health emergencies should move beyond pathogen control alone. The core implication of the literature is that epidemic governance should be understood not simply as a biomedical response but also as a problem of social resilience. Countries with stronger levels of social trust, more legitimate institutions, and more effective state capacity generally performed better during the COVID-19 pandemic than conventional preparedness indices alone predicted (7, 8). The sustained public support was related to trust, clear communication, shared identity, and the perception that policies are fair and collectively beneficial (9, 10). These findings suggest that social resilience is not a secondary outcome of successful epidemic management but one of its preconditions.

The first implication is that risk communication should be treated as a core intervention. During prolonged crises, communication needs to do more than simply convey threats. It should also sustain agency, legitimacy, and a workable sense of future orientation. Hope-oriented public communication can motivate compliance more effectively than fear-based messaging alone during prolonged pandemic conditions can (75). Credible communication depends on clarity, consistency, transparency about uncertainty, and the avoidance of false reassurance (76). This finding implies that authorities should communicate not only risks but also what people can do, why those actions are important, and how collective sacrifice relates to plausible public benefits. Effective communication therefore serves a governance function by sustaining trust, reducing uncertainty, and preventing the amplification of crisis pressures.

The second implication is that infodemic management should be integrated into routine outbreak response. Studies focused on COVID-19 have shown that information disorders can influence risk perception, anxiety, compliance, and polarization in ways that materially affect crisis trajectories. Most of the infodemic interventions used during the COVID-19 pandemic involved social listening, evidence-based communication, digital tools, and coordinated content responses, but the implementation evidence remains limited in quality (74). In the next phase of research and policy, broader information ecosystems—such as overload, information gaps, competing interpretations, and platform dynamics—need to be investigated (71). This gap suggests that effective epidemic governance requires institutional capacity more than merely correcting falsehoods. Managing the information environment means reducing feedback loops that reinforce uncertainty, distrust, and behavioral escalation.

Social protection should also be treated as a public health measure. Financial strain, unequal exposure, and disrupted access to work, food, and care have been shown to be central drivers of psychological burden and social grievances. This understanding means that interventions such as income replacement, food support, housing security, and access to routine care are important for preventing behavioral destabilization and preserving trust. New data systems can improve the targeting of aid in crisis settings in which conventional registries are weak, highlighting the value of rapid social assistance mechanisms during shocks (77).

Institutions should take active measures to maintain cross-cutting social connections and mitigate polarization during crises. Pandemic conditions can intensify homophily, stigma, and moralized divisions, especially when communication becomes conflictual and social interaction shifts to more selective digital environments (67, 73). Strategies that rely exclusively on top-down instruction might be less effective when trust is weak or when information is filtered through politicized group identities. Community-engaged approaches, trusted intermediaries, and communication via locally credible networks might be more important in settings with inequality or prior mistrust (9, 10).

## Conclusion

Public health emergencies are not only epidemiological events but also social crises whose dynamics are shaped by interactions among social consequences, trust dynamics, behavioral responses, and information environments. This review argues that the crisis dimensions of epidemics are continuously amplified, sustained, or attenuated through interconnected social processes. Epidemics interact with existing inequalities in livelihoods, education, healthcare access, and social protection, producing uneven burdens across populations and influencing how crises are experienced and governed.

A central contribution of this review is the proposal of an integrated mechanism framework that links social consequences, trust, behavioral and psychological responses, and information environments within a single analytical perspective. We conclude that trust is the main mediator in this process. Trust influences how people interpret risk, whether they accept collective restrictions, and how they respond to official communication under uncertainty. When trust weakens, social disruption is more likely to be processed through suspicion, grievance, and noncompliance. Moreover, behavioral and psychological responses such as anxiety, loneliness, fear, and information overload can amplify instability, especially when they are intensified by digital misinformation, stigma, and polarization.

The key implication is that epidemic preparedness must move beyond a pathogen-centered model. Surveillance and clinical capacity remain essential, but they are insufficient if communication is inconsistent, social support is weak, and trust is allowed to deteriorate. Therefore, future preparedness efforts should integrate public health responses with social protection, mental health support, trust-sensitive communication, and infodemic management. If public health systems are to prevent future outbreaks from escalating into wider social crises, they should be designed in a way that considers both disease control and social resilience under prolonged conditions of uncertainty.
